# Supplementary material for: Hot spot prediction in protein-protein interactions by an ensemble system
Source: BMC Syst Biol. 2018 Dec 31;12(Suppl 9):132. doi: 10.1186/s12918-018-0665-8 (PMC6311905; doi:10.1186/s12918-018-0665-8)
Supplement: Supplementary file 2 — All relevant MATLAB code. A simple Matlab implement of our predictor. (ZIP 19,742 kb) [file 12918_2018_665_MOESM2_ESM.zip › Additional file 2/Read ASA/ASA Property/SKEMPI/2GOXB.txt]

 NetSurfP - Protein Surface Accessibility and Secondary Structure Predictions

  

|  |  |  |
| --- | --- | --- |
|  |  | NetSurfP - Protein Surface Accessibility and Secondary Structure Predictions  Technical University of Denmark |

  


---

```
# For publication of results, please cite:
# A generic method for assignment of reliability scores applied to solvent accessibility predictions.
# Bent Petersen, Thomas Nordahl Petersen, Pernille Andersen, Morten Nielsen and Claus Lundegaard
# BMC Structural Biology 2009, 9:51 doi:10.1186/1472-6807-9-51
#
# Column 1: Class assignment - B for buried or E for Exposed - Threshold: 25% exposure, but not based on RSA
# Column 2: Amino acid
# Column 3: Sequence name
# Column 4: Amino acid number
# Column 5: Relative Surface Accessibility - RSA
# Column 6: Absolute Surface Accessibility
# Column 7: Z-fit score for RSA prediction
# Column 8: Probability for Alpha-Helix
# Column 9: Probability for Beta-strand
# Column 10: Probability for Coil
E T  2GOXB                  1    0.844 117.049  -0.016   0.003   0.003   0.994
E D  2GOXB                  2    0.677  97.527  -1.129   0.257   0.016   0.727
E A  2GOXB                  3    0.502  55.320  -0.992   0.522   0.016   0.462
E T  2GOXB                  4    0.517  71.722   0.396   0.622   0.015   0.363
B I  2GOXB                  5    0.216  39.868   0.172   0.717   0.014   0.269
E K  2GOXB                  6    0.526 108.178   1.108   0.802   0.014   0.185
E K  2GOXB                  7    0.508 104.578   0.610   0.879   0.010   0.111
E E  2GOXB                  8    0.397  69.391  -0.338   0.879   0.010   0.111
E Q  2GOXB                  9    0.494  88.300  -0.022   0.879   0.010   0.111
E K  2GOXB                 10    0.484  99.518   0.615   0.879   0.010   0.111
B L  2GOXB                 11    0.195  35.778   0.271   0.879   0.010   0.111
B I  2GOXB                 12    0.244  45.196  -0.065   0.938   0.007   0.055
E Q  2GOXB                 13    0.500  89.336   0.370   0.938   0.007   0.055
B A  2GOXB                 14    0.140  15.439  -1.053   0.923   0.002   0.076
E Q  2GOXB                 15    0.426  76.173   0.163   0.858   0.002   0.139
E N  2GOXB                 16    0.530  77.563  -0.144   0.858   0.002   0.139
B L  2GOXB                 17    0.137  25.103   0.675   0.879   0.010   0.111
B V  2GOXB                 18    0.126  19.397  -0.327   0.938   0.007   0.055
E R  2GOXB                 19    0.513 117.431   0.869   0.879   0.010   0.111
E E  2GOXB                 20    0.512  89.446   0.723   0.879   0.010   0.111
B F  2GOXB                 21    0.098  19.729   0.238   0.802   0.014   0.185
E E  2GOXB                 22    0.561  98.024   0.311   0.717   0.014   0.269
E K  2GOXB                 23    0.619 127.431   0.361   0.660   0.049   0.291
E T  2GOXB                 24    0.407  56.409  -0.187   0.502   0.102   0.396
E H  2GOXB                 25    0.337  61.209  -0.084   0.307   0.165   0.527
E T  2GOXB                 26    0.412  57.186  -0.087   0.321   0.252   0.427
B V  2GOXB                 27    0.237  36.396  -0.348   0.321   0.252   0.427
E S  2GOXB                 28    0.502  58.811  -0.410   0.538   0.173   0.289
E A  2GOXB                 29    0.303  33.435  -0.742   0.660   0.049   0.291
E H  2GOXB                 30    0.336  61.191  -0.585   0.660   0.049   0.291
E R  2GOXB                 31    0.462 105.867   0.104   0.751   0.050   0.199
E K  2GOXB                 32    0.513 105.462   0.329   0.751   0.050   0.199
B A  2GOXB                 33    0.173  19.032  -1.337   0.751   0.050   0.199
E Q  2GOXB                 34    0.487  86.960  -0.015   0.660   0.049   0.291
E K  2GOXB                 35    0.544 111.963   0.481   0.701   0.107   0.192
B A  2GOXB                 36    0.226  24.850  -0.754   0.605   0.105   0.290
B V  2GOXB                 37    0.208  32.016  -0.236   0.453   0.248   0.299
E N  2GOXB                 38    0.505  73.917  -0.183   0.231   0.330   0.439
B L  2GOXB                 39    0.200  36.675   0.086   0.252   0.423   0.325
B V  2GOXB                 40    0.222  34.075  -0.189   0.148   0.418   0.435
E S  2GOXB                 41    0.406  47.607  -0.529   0.252   0.423   0.325
B F  2GOXB                 42    0.201  40.341   0.112   0.373   0.401   0.226
E E  2GOXB                 43    0.507  88.503   0.149   0.478   0.309   0.214
E Y  2GOXB                 44    0.276  58.938   0.419   0.594   0.285   0.121
E K  2GOXB                 45    0.456  93.717   0.403   0.662   0.225   0.113
B V  2GOXB                 46    0.152  23.424  -0.164   0.725   0.163   0.112
E K  2GOXB                 47    0.388  79.853   0.274   0.779   0.100   0.120
E K  2GOXB                 48    0.436  89.747   0.255   0.831   0.044   0.125
E M  2GOXB                 49    0.327  65.493   0.488   0.911   0.033   0.057
B V  2GOXB                 50    0.197  30.325   0.273   0.879   0.010   0.111
B L  2GOXB                 51    0.131  24.078   0.410   0.938   0.007   0.055
E Q  2GOXB                 52    0.427  76.262   0.751   0.938   0.007   0.055
E E  2GOXB                 53    0.613 107.178   0.404   0.938   0.007   0.055
E R  2GOXB                 54    0.328  75.158   0.355   0.923   0.002   0.076
B I  2GOXB                 55    0.067  12.339  -0.176   0.858   0.002   0.139
E D  2GOXB                 56    0.547  78.866   0.314   0.923   0.002   0.076
E N  2GOXB                 57    0.520  76.157   0.364   0.923   0.002   0.076
B V  2GOXB                 58    0.128  19.674  -0.191   0.923   0.002   0.076
B L  2GOXB                 59    0.180  32.903   0.178   0.923   0.002   0.076
E K  2GOXB                 60    0.599 123.153   0.656   0.858   0.002   0.139
E Q  2GOXB                 61    0.571 101.927   1.111   0.694   0.003   0.303
E G  2GOXB                 62    0.402  31.598  -1.711   0.502   0.002   0.495
E L  2GOXB                 63    0.360  65.824  -0.440   0.339   0.016   0.645
E V  2GOXB                 64    0.519  79.693  -0.704   0.181   0.016   0.803
E R  2GOXB                 65    0.817 187.047   0.758   0.003   0.003   0.994

Explain the output.  Go back.
```

---
